# A New Pronouncing Dictionary of Medicine

**Published:** 1893-06

**Authors:** 


*A New Pronouncing Dictionary of Medicine.* By JOHN M. KEATING, M.D. AND HENRY HAMILTON. Pp. 318. Edinburgh: YOUNG J. PENTLAND. 1892.

The multiplication of reference books should be enthusiastically encouraged. In each we find something which the others have not; there is fresh material or new arrangement which will make it a desirable companion. General literature and special subjects increasingly need these books, for there is a perpetual broadening out of all departments of knowledge. Life is being lived at such a pace that for many things we have time only to refer to an authoritative summary of the existing knowledge concerning them; and as new phases of inquiry, with additions to scientific terminology, are being presented almost daily, it is imperative that books of the nature of the one before us should be constantly renewed.

Although doctors had no small stock of medical dictionaries, we are sure there is room for this one, which has been compiled chiefly by Dr. Keating, favourably known to English readers as the editor of the *Cyclopædia of the Diseases of Children.* Although on the difficult question of the pronunciation of English words formed from Greek and Latin originals there will always be a considerable variety of opinion, his lead may generally be followed without much damage resulting. It is impossible at present to lay down special rules thereon which will be universally accepted, and this is made abundantly clear from the replies which were received from the scholars who were appealed to in reference to the preparation of this book.

In addition to the dictionary part, there is in the volume other matter covering nearly a hundred pages, and full of interesting information, presented in a useful form, and of so varied a character as to extend from weights and measures and scales to abstruse details, anatomical and pathological.

Altogether, the book is one to have and to use. Its type and arrangement are excellent.

